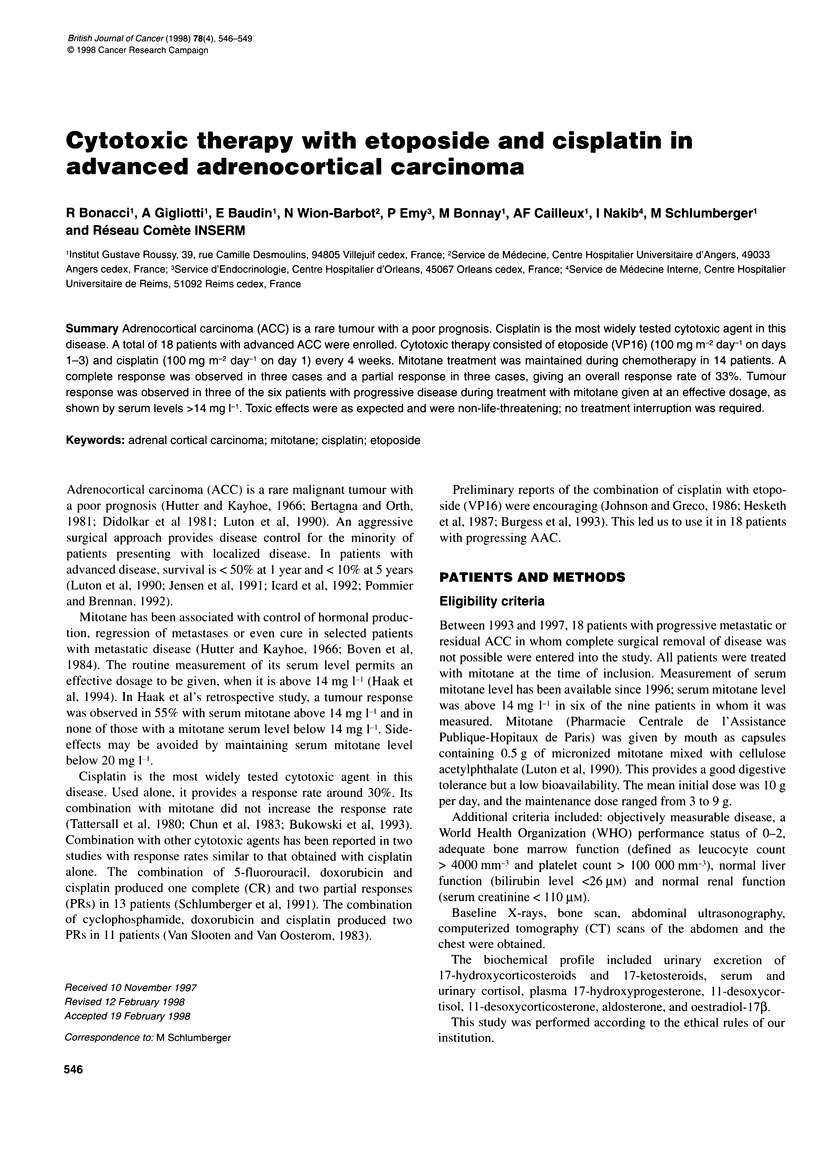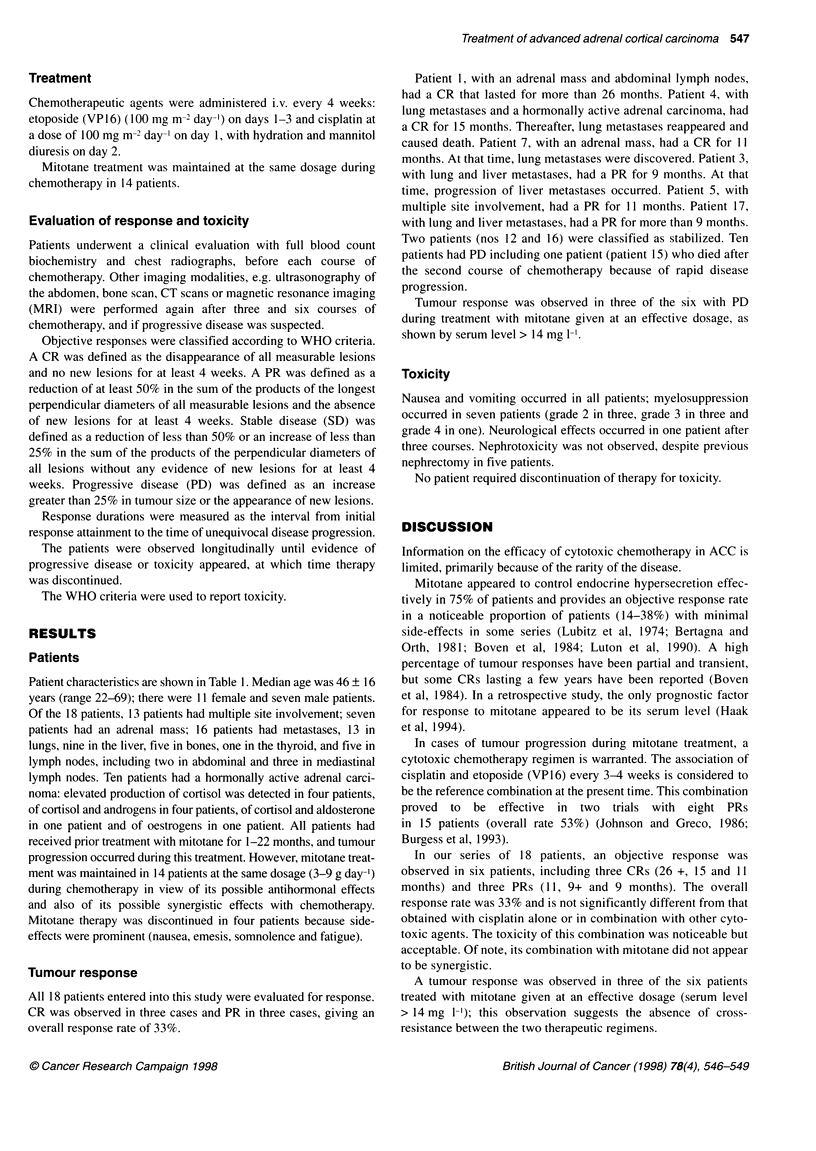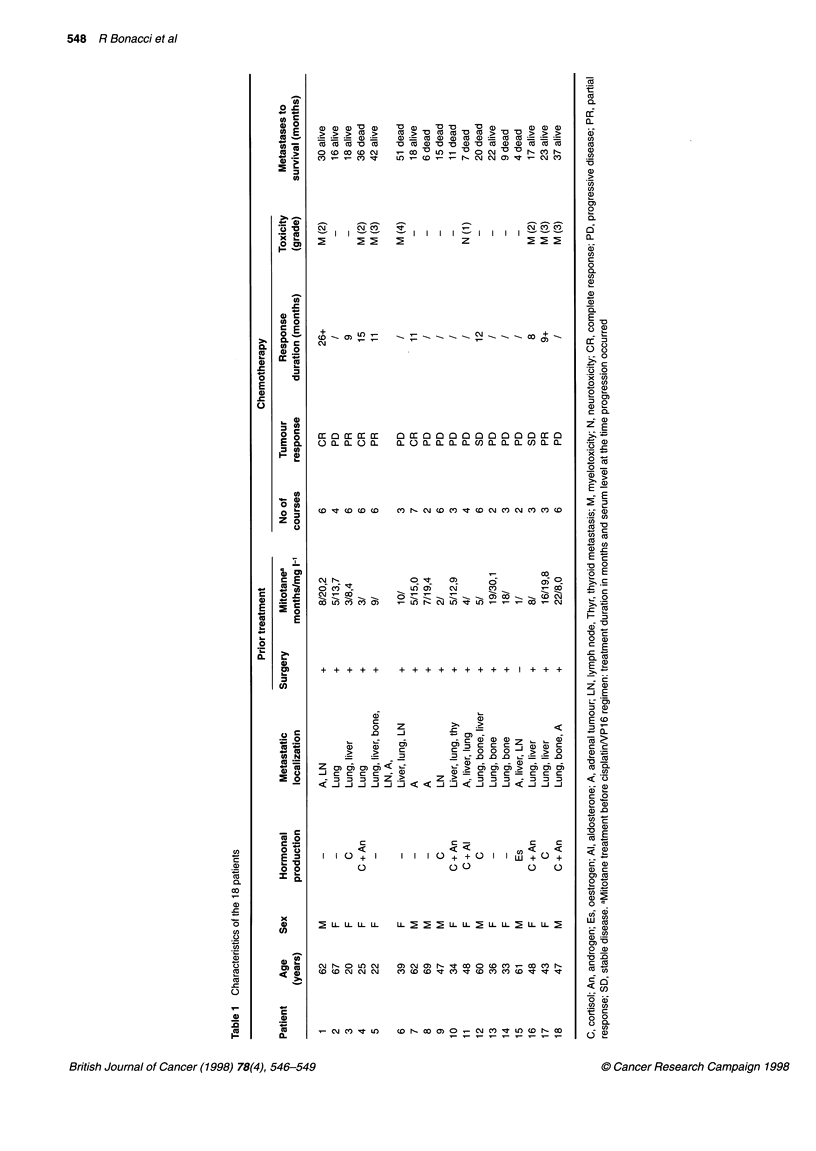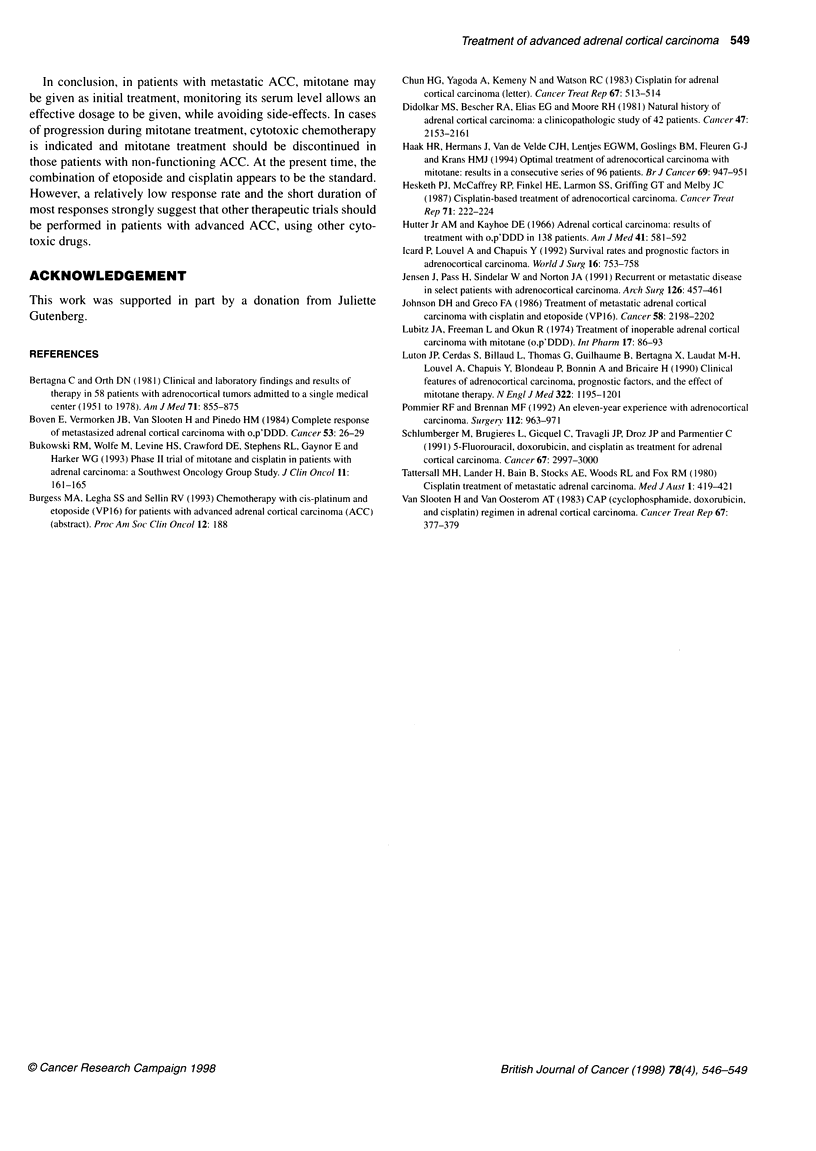# Cytotoxic therapy with etoposide and cisplatin in advanced adrenocortical carcinoma. Réseau Comète INSERM.

**DOI:** 10.1038/bjc.1998.530

**Published:** 1998-08

**Authors:** R. Bonacci, A. Gigliotti, E. Baudin, N. Wion-Barbot, P. Emy, M. Bonnay, A. F. Cailleux, I. Nakib, M. Schlumberger

**Affiliations:** Institut Gustave Roussy, Villejuif, France.

## Abstract

Adrenocortical carcinoma (ACC) is a rare tumour with a poor prognosis. Cisplatin is the most widely tested cytotoxic agent in this disease. A total of 18 patients with advanced ACC were enrolled. Cytotoxic therapy consisted of etoposide (VP16) (100 mg m(-2) day(-1) on days 1-3) and cisplatin (100 mg m(-2) day(-1) on day 1) every 4 weeks. Mitotane treatment was maintained during chemotherapy in 14 patients. A complete response was observed in three cases and a partial response in three cases, giving an overall response rate of 33%. Tumour response was observed in three of the six patients with progressive disease during treatment with mitotane given at an effective dosage, as shown by serum levels >14 mg l(-1). Toxic effects were as expected and were non-life-threatening; no treatment interruption was required.


British Journal of Cancer (1998) 78(4), 546–549
© 1998 Cancer Research Campaign

### Cytotoxic therapy with etoposide and cisplatin in advanced adrenocortical carcinoma

**R Bonacci[1], A Gigliotti[1], E Baudin[1], N Wion-Barbot[2], P Emy[3], M Bonnay[1], AF Cailleux[1], I Nakib[4], M Schlumberger[1] and Réseau Comète INSERM**

[1]Institut Gustave Roussy, 39, rue Camille Desmoulins, 94805 Villejuif cedex, France; [2]Service de Médecine, Centre Hospitalier Universitaire d'Angers, 49033 Angers cedex, France; [3]Service d'Endocrinologie, Centre Hospitalier d'Orleans, 45067 Orleans cedex, France; [4]Service de Médecine Interne, Centre Hospitalier Universitaire de Reims, 51092 Reims cedex, France

**Summary** Adrenocortical carcinoma (ACC) is a rare tumour with a poor prognosis. Cisplatin is the most widely tested cytotoxic agent in this disease. A total of 18 patients with advanced ACC were enrolled. Cytotoxic therapy consisted of etoposide (VP16) (100 mg $m^{-2}$ $day^{-1}$ on days 1–3) and cisplatin (100 mg $m^{-2}$ $day^{-1}$ on day 1) every 4 weeks. Mitotane treatment was maintained during chemotherapy in 14 patients. A complete response was observed in three cases and a partial response in three cases, giving an overall response rate of 33%. Tumour response was observed in three of the six patients with progressive disease during treatment with mitotane given at an effective dosage, as shown by serum levels >14 mg $l^{-1}$. Toxic effects were as expected and were non-life-threatening; no treatment interruption was required.

**Keywords:** adrenal cortical carcinoma; mitotane; cisplatin; etoposide

Adrenocortical carcinoma (ACC) is a rare malignant tumour with a poor prognosis (Hutter and Kayhoe, 1966; Bertagna and Orth, 1981; Didolkar et al 1981; Luton et al, 1990). An aggressive surgical approach provides disease control for the minority of patients presenting with localized disease. In patients with advanced disease, survival is < 50% at 1 year and < 10% at 5 years (Luton et al, 1990; Jensen et al, 1991; Icard et al, 1992; Pommier and Brennan, 1992).

Mitotane has been associated with control of hormonal production, regression of metastases or even cure in selected patients with metastatic disease (Hutter and Kayhoe, 1966; Boven et al, 1984). The routine measurement of its serum level permits an effective dosage to be given, when it is above 14 mg $l^{-1}$ (Haak et al, 1994). In Haak et al's retrospective study, a tumour response was observed in 55% with serum mitotane above 14 mg $l^{-1}$ and in none of those with a mitotane serum level below 14 mg $l^{-1}$. Side-effects may be avoided by maintaining serum mitotane level below 20 mg $l^{-1}$.

Cisplatin is the most widely tested cytotoxic agent in this disease. Used alone, it provides a response rate around 30%. Its combination with mitotane did not increase the response rate (Tattersall et al, 1980; Chun et al, 1983; Bukowski et al, 1993). Combination with other cytotoxic agents has been reported in two studies with response rates similar to that obtained with cisplatin alone. The combination of 5-fluorouracil, doxorubicin and cisplatin produced one complete (CR) and two partial responses (PRs) in 13 patients (Schlumberger et al, 1991). The combination of cyclophosphamide, doxorubicin and cisplatin produced two PRs in 11 patients (Van Slooten and Van Oosterom, 1983).

*Received 10 November 1997*
*Revised 12 February 1998*
*Accepted 19 February 1998*

*Correspondence to:* M Schlumberger

Preliminary reports of the combination of cisplatin with etoposide (VP16) were encouraging (Johnson and Greco, 1986; Hesketh et al, 1987; Burgess et al, 1993). This led us to use it in 18 patients with progressing AAC.

### PATIENTS AND METHODS

### Eligibility criteria

Between 1993 and 1997, 18 patients with progressive metastatic or residual ACC in whom complete surgical removal of disease was not possible were entered into the study. All patients were treated with mitotane at the time of inclusion. Measurement of serum mitotane level has been available since 1996; serum mitotane level was above 14 mg $l^{-1}$ in six of the nine patients in whom it was measured. Mitotane (Pharmacie Centrale de l'Assistance Publique-Hopitaux de Paris) was given by mouth as capsules containing 0.5 g of micronized mitotane mixed with cellulose acetylphthalate (Luton et al, 1990). This provides a good digestive tolerance but a low bioavailability. The mean initial dose was 10 g per day, and the maintenance dose ranged from 3 to 9 g.

Additional criteria included: objectively measurable disease, a World Health Organization (WHO) performance status of 0–2, adequate bone marrow function (defined as leucocyte count > 4000 $mm^{-3}$ and platelet count > 100 000 $mm^{-3}$), normal liver function (bilirubin level <26 μM) and normal renal function (serum creatinine < 110 μM).

Baseline X-rays, bone scan, abdominal ultrasonography, computerized tomography (CT) scans of the abdomen and the chest were obtained.

The biochemical profile included urinary excretion of 17-hydroxycorticosteroids and 17-ketosteroids, serum and urinary cortisol, plasma 17-hydroxyprogesterone, 11-desoxycortisol, 11-desoxycorticosterone, aldosterone, and oestradiol-17β.

This study was performed according to the ethical rules of our institution.

### Treatment

Chemotherapeutic agents were administered i.v. every 4 weeks: etoposide (VP16) (100 mg $m^{-2}$ $day^{-1}$) on days 1–3 and cisplatin at a dose of 100 mg $m^{-2}$ $day^{-1}$ on day 1, with hydration and mannitol diuresis on day 2.

Mitotane treatment was maintained at the same dosage during chemotherapy in 14 patients.

### Evaluation of response and toxicity

Patients underwent a clinical evaluation with full blood count biochemistry and chest radiographs, before each course of chemotherapy. Other imaging modalities, e.g. ultrasonography of the abdomen, bone scan, CT scans or magnetic resonance imaging (MRI) were performed again after three and six courses of chemotherapy, and if progressive disease was suspected.

Objective responses were classified according to WHO criteria. A CR was defined as the disappearance of all measurable lesions and no new lesions for at least 4 weeks. A PR was defined as a reduction of at least 50% in the sum of the products of the longest perpendicular diameters of all measurable lesions and the absence of new lesions for at least 4 weeks. Stable disease (SD) was defined as a reduction of less than 50% or an increase of less than 25% in the sum of the products of the perpendicular diameters of all lesions without any evidence of new lesions for at least 4 weeks. Progressive disease (PD) was defined as an increase greater than 25% in tumour size or the appearance of new lesions.

Response durations were measured as the interval from initial response attainment to the time of unequivocal disease progression.

The patients were observed longitudinally until evidence of progressive disease or toxicity appeared, at which time therapy was discontinued.

The WHO criteria were used to report toxicity.

### RESULTS

### Patients

Patient characteristics are shown in Table 1. Median age was 46 ± 16 years (range 22–69); there were 11 female and seven male patients. Of the 18 patients, 13 patients had multiple site involvement; seven patients had an adrenal mass; 16 patients had metastases, 13 in lungs, nine in the liver, five in bones, one in the thyroid, and five in lymph nodes, including two in abdominal and three in mediastinal lymph nodes. Ten patients had a hormonally active adrenal carcinoma: elevated production of cortisol was detected in four patients, of cortisol and androgens in four patients, of cortisol and aldosterone in one patient and of oestrogens in one patient. All patients had received prior treatment with mitotane for 1–22 months, and tumour progression occurred during this treatment. However, mitotane treatment was maintained in 14 patients at the same dosage (3–9 g $day^{-1}$) during chemotherapy in view of its possible antihormonal effects and also of its possible synergistic effects with chemotherapy. Mitotane therapy was discontinued in four patients because side-effects were prominent (nausea, emesis, somnolence and fatigue).

### Tumour response

All 18 patients entered into this study were evaluated for response. CR was observed in three cases and PR in three cases, giving an overall response rate of 33%.

Patient 1, with an adrenal mass and abdominal lymph nodes, had a CR that lasted for more than 26 months. Patient 4, with lung metastases and a hormonally active adrenal carcinoma, had a CR for 15 months. Thereafter, lung metastases reappeared and caused death. Patient 7, with an adrenal mass, had a CR for 11 months. At that time, lung metastases were discovered. Patient 3, with lung and liver metastases, had a PR for 9 months. At that time, progression of liver metastases occurred. Patient 5, with multiple site involvement, had a PR for 11 months. Patient 17, with lung and liver metastases, had a PR for more than 9 months. Two patients (nos 12 and 16) were classified as stabilized. Ten patients had PD including one patient (patient 15) who died after the second course of chemotherapy because of rapid disease progression.

Tumour response was observed in three of the six with PD during treatment with mitotane given at an effective dosage, as shown by serum level > 14 mg $l^{-1}$.

### Toxicity

Nausea and vomiting occurred in all patients; myelosuppression occurred in seven patients (grade 2 in three, grade 3 in three and grade 4 in one). Neurological effects occurred in one patient after three courses. Nephrotoxicity was not observed, despite previous nephrectomy in five patients.

No patient required discontinuation of therapy for toxicity.

### DISCUSSION

Information on the efficacy of cytotoxic chemotherapy in ACC is limited, primarily because of the rarity of the disease.

Mitotane appeared to control endocrine hypersecretion effectively in 75% of patients and provides an objective response rate in a noticeable proportion of patients (14–38%) with minimal side-effects in some series (Lubitz et al, 1974; Bertagna and Orth, 1981; Boven et al, 1984; Luton et al, 1990). A high percentage of tumour responses have been partial and transient, but some CRs lasting a few years have been reported (Boven et al, 1984). In a retrospective study, the only prognostic factor for response to mitotane appeared to be its serum level (Haak et al, 1994).

In cases of tumour progression during mitotane treatment, a cytotoxic chemotherapy regimen is warranted. The association of cisplatin and etoposide (VP16) every 3–4 weeks is considered to be the reference combination at the present time. This combination proved to be effective in two trials with eight PRs in 15 patients (overall rate 53%) (Johnson and Greco, 1986; Burgess et al, 1993).

In our series of 18 patients, an objective response was observed in six patients, including three CRs (26 +, 15 and 11 months) and three PRs (11, 9+ and 9 months). The overall response rate was 33% and is not significantly different from that obtained with cisplatin alone or in combination with other cytotoxic agents. The toxicity of this combination was noticeable but acceptable. Of note, its combination with mitotane did not appear to be synergistic.

A tumour response was observed in three of the six patients treated with mitotane given at an effective dosage (serum level > 14 mg $l^{-1}$); this observation suggests the absence of cross-resistance between the two therapeutic regimens.

© Cancer Research Campaign 1998

**Table 1** Characteristics of the 18 patients

| Patient | Age (years) | Sex | Hormonal production | Metastatic localization | Prior treatment: Surgery | Prior treatment: Mitotane[a] months/mg $l^{-1}$ | Chemotherapy: No of courses | Chemotherapy: Tumour response | Chemotherapy: Response duration (months) | Chemotherapy: Toxicity (grade) | Metastases to survival (months) |
|---|---|---|---|---|---|---|---|---|---|---|---|
| 1 | 62 | M | – | A, LN | + | 8/20,2 | 6 | CR | 26+ | M (2) | 30 alive |
| 2 | 67 | F | – | Lung | + | 5/13,7 | 4 | PD | / | – | 16 alive |
| 3 | 20 | F | C | Lung, liver | + | 3/8,4 | 6 | PR | 9 | – | 18 alive |
| 4 | 25 | F | C + An | Lung | + | 3/ | 6 | CR | 15 | M (2) | 36 dead |
| 5 | 22 | F | – | Lung, liver, bone, LN, A, | + | 9/ | 6 | PR | 11 | M (3) | 42 alive |
| 6 | 39 | F | – | Liver, lung, LN | + | 10/ | 3 | PD | / | M (4) | 51 dead |
| 7 | 62 | M | – | A | + | 5/15,0 | 7 | CR | 11 | – | 18 alive |
| 8 | 69 | M | – | A | + | 7/19,4 | 2 | PD | / | – | 6 dead |
| 9 | 47 | M | C | LN | + | 2/ | 6 | PD | / | – | 15 dead |
| 10 | 34 | F | C + An | Liver, lung, thy | + | 5/12,9 | 3 | PD | / | – | 11 dead |
| 11 | 48 | F | C + Al | A, liver, lung | + | 4/ | 4 | PD | / | N (1) | 7 dead |
| 12 | 60 | M | C | Lung, bone, liver | + | 5/ | 6 | SD | 12 | – | 20 dead |
| 13 | 36 | F | – | Lung, bone | + | 19/30,1 | 2 | PD | / | – | 22 alive |
| 14 | 33 | F | – | Lung, bone | + | 18/ | 3 | PD | / | – | 9 dead |
| 15 | 61 | M | Es | A, liver, LN | – | 1/ | 2 | PD | / | – | 4 dead |
| 16 | 48 | F | C + An | Lung, liver | + | 8/ | 3 | SD | 8 | M (2) | 17 alive |
| 17 | 43 | F | C | Lung, liver | + | 16/19,8 | 3 | PR | 9+ | M (3) | 23 alive |
| 18 | 47 | M | C + An | Lung, bone, A | + | 22/8,0 | 6 | PD | / | M (3) | 37 alive |

C, cortisol; An, androgen; Es, oestrogen; Al, aldosterone; A, adrenal tumour; LN, lymph node, Thyr, thyroid metastasis; M, myelotoxicity; N, neurotoxicity; CR, complete response; PD, progressive disease; PR, partial response; SD, stable disease. [a]Mitotane treatment before cisplatin/VP16 regimen: treatment duration in months and serum level at the time progression occurred

*© Cancer Research Campaign 1998*

In conclusion, in patients with metastatic ACC, mitotane may be given as initial treatment, monitoring its serum level allows an effective dosage to be given, while avoiding side-effects. In cases of progression during mitotane treatment, cytotoxic chemotherapy is indicated and mitotane treatment should be discontinued in those patients with non-functioning ACC. At the present time, the combination of etoposide and cisplatin appears to be the standard. However, a relatively low response rate and the short duration of most responses strongly suggest that other therapeutic trials should be performed in patients with advanced ACC, using other cytotoxic drugs.

### ACKNOWLEDGEMENT

This work was supported in part by a donation from Juliette Gutenberg.

### REFERENCES

Bertagna C and Orth DN (1981) Clinical and laboratory findings and results of therapy in 58 patients with adrenocortical tumors admitted to a single medical center (1951 to 1978). *Am J Med* **71**: 855–875

Boven E, Vermorken JB, Van Slooten H and Pinedo HM (1984) Complete response of metastasized adrenal cortical carcinoma with o,p'DDD. *Cancer* **53**: 26–29

Bukowski RM, Wolfe M, Levine HS, Crawford DE, Stephens RL, Gaynor E and Harker WG (1993) Phase II trial of mitotane and cisplatin in patients with adrenal carcinoma: a Southwest Oncology Group Study. *J Clin Oncol* **11**: 161–165

Burgess MA, Legha SS and Sellin RV (1993) Chemotherapy with cis-platinum and etoposide (VP16) for patients with advanced adrenal cortical carcinoma (ACC) (abstract). *Proc Am Soc Clin Oncol* **12**: 188

Chun HG, Yagoda A, Kemeny N and Watson RC (1983) Cisplatin for adrenal cortical carcinoma (letter). *Cancer Treat Rep* **67**: 513–514

Didolkar MS, Bescher RA, Elias EG and Moore RH (1981) Natural history of adrenal cortical carcinoma: a clinicopathologic study of 42 patients. *Cancer* **47**: 2153–2161

Haak HR, Hermans J, Van de Velde CJH, Lentjes EGWM, Goslings BM, Fleuren G-J and Krans HMJ (1994) Optimal treatment of adrenocortical carcinoma with mitotane: results in a consecutive series of 96 patients. *Br J Cancer* **69**: 947–951

Hesketh PJ, McCaffrey RP, Finkel HE, Larmon SS, Griffing GT and Melby JC (1987) Cisplatin-based treatment of adrenocortical carcinoma. *Cancer Treat Rep* **71**: 222–224

Hutter Jr AM and Kayhoe DE (1966) Adrenal cortical carcinoma: results of treatment with o,p'DDD in 138 patients. *Am J Med* **41**: 581–592

Icard P, Louvel A and Chapuis Y (1992) Survival rates and prognostic factors in adrenocortical carcinoma. *World J Surg* **16**: 753–758

Jensen J, Pass H, Sindelar W and Norton JA (1991) Recurrent or metastatic disease in select patients with adrenocortical carcinoma. *Arch Surg* **126**: 457–461

Johnson DH and Greco FA (1986) Treatment of metastatic adrenal cortical carcinoma with cisplatin and etoposide (VP16). *Cancer* **58**: 2198–2202

Lubitz JA, Freeman L and Okun R (1974) Treatment of inoperable adrenal cortical carcinoma with mitotane (o,p'DDD). *Int Pharm* **17**: 86–93

Luton JP, Cerdas S, Billaud L, Thomas G, Guilhaume B, Bertagna X, Laudat M-H, Louvel A, Chapuis Y, Blondeau P, Bonnin A and Bricaire H (1990) Clinical features of adrenocortical carcinoma, prognostic factors, and the effect of mitotane therapy. *N Engl J Med* **322**: 1195–1201

Pommier RF and Brennan MF (1992) An eleven-year experience with adrenocortical carcinoma. *Surgery* **112**: 963–971

Schlumberger M, Brugieres L, Gicquel C, Travagli JP, Droz JP and Parmentier C (1991) 5-Fluorouracil, doxorubicin, and cisplatin as treatment for adrenal cortical carcinoma. *Cancer* **67**: 2997–3000

Tattersall MH, Lander H, Bain B, Stocks AE, Woods RL and Fox RM (1980) Cisplatin treatment of metastatic adrenal carcinoma. *Med J Aust* **1**: 419–421

Van Slooten H and Van Oosterom AT (1983) CAP (cyclophosphamide, doxorubicin, and cisplatin) regimen in adrenal cortical carcinoma. *Cancer Treat Rep* **67**: 377–379

© Cancer Research Campaign 1998